# Beyond light’s criteria: pleural lactate predicts need for tube thoracostomy in complicated parapneumonic effusions

**DOI:** 10.1007/s11845-026-04435-7

**Published:** 2026-05-06

**Authors:** Mahmut Ozbey, Ibrahim Can Kürkçüoğlu

**Affiliations:** 1 Department of Thoracic Surgery, Sivas Cumhuriyet University, Faculty of Medicine, Sivas, Turkey; 2 Department of Thoracic Surgery, Liv Hospital Gaziantep, Gaziantep, Turkey

**Keywords:** Exudate, Lactate, Parapneumonic effusion, Pleural effusion, Tube thoracostomy

## Abstract

**Background:**

Differentiation of pleural effusions and identification of complicated parapneumonic effusions remain important challenges in clinical practice. Although Light’s criteria are routinely applied, additional pleural fluid parameters may provide complementary information in selected patients.

**Aims:**

To evaluate the diagnostic performance of pleural fluid lactate and the pleural fluid-to-blood lactate ratio in pleural effusions and to examine their association with complicated parapneumonic effusions and drainage approach.

**Methods:**

This study included 110 patients who underwent pleural drainage. Paired pleural fluid and venous blood samples were obtained prior to drainage, and lactate levels were measured using a blood gas analyser. Pleural effusions were classified according to Light’s criteria. Diagnostic performance was assessed using receiver operating characteristic analysis.

**Results:**

Of the 110 patients, 90 (81.8%) had exudative and 20 (18.2%) transudative effusions. Pleural fluid lactate and the pleural fluid-to-blood lactate ratio showed good discrimination between exudative and transudative effusions (AUC 0.912 and 0.883, *p* < 0.001), while blood lactate alone had limited diagnostic value. No significant correlation was observed between pleural and blood lactate levels. In patients with complicated parapneumonic effusions, higher pleural fluid lactate levels were associated with complicated effusions (cut-off 5.45 mmol/L; AUC 0.883). Elevated pleural fluid lactate values were also more frequently observed in patients who required tube thoracostomy (cut-off 4.05 mmol/L; AUC 0.920).

**Conclusions:**

Pleural fluid lactate may serve as a useful adjunct to standard pleural fluid analysis. Its measurement could assist in the assessment of disease severity and drainage requirements in parapneumonic effusions; however, further prospective studies are warranted.

## Introduction

Pleural effusion, the pathological accumulation of fluid in the pleural space, is a common clinical manifestation of a wide spectrum of diseases, ranging from cardiac failure and cirrhosis to malignancies and infections [1]. The initial and crucial step in the diagnostic algorithm of pleural effusion is the differentiation between transudates and exudates, as this distinction fundamentally guides subsequent etiological investigation and management [2]. Light’s criteria, which utilize pleural fluid and serum protein and lactate dehydrogenase (LDH) levels, have been the cornerstone for this differentiation for decades [3–5]. However, the quest for more rapid, accessible, and equally accurate biomarkers is ongoing.

In recent years, the role of pleural fluid lactate (PFL) has garnered significant interest. Lactate, a product of anaerobic glycolysis and cellular metabolism, is postulated to reflect the inflammatory and metabolic activity within the pleural space. Elevated PFL levels are theorized to occur in exudative effusions due to the heightened metabolic activity of immune cells, bacterial metabolism, and possibly impaired clearance [6, 7]. Consequently, PFL has been investigated not only for the initial transudate-exudate separation but also for risk stratification within specific exudative categories, particularly parapneumonic effusions (PPE), where distinguishing between simple and complicated cases is critical for determining the need for invasive drainage procedures [7, 8].

Despite promising findings, the existing literature presents variable cut-off values for PFL and conflicting evidence regarding the influence of blood lactate (BL) on PFL levels. Some studies suggest that the gradient between PFL and BL may be a more reliable parameter, while others propose that PFL operates independently of systemic lactate concentrations [6, 9]. This ambiguity underscores the need for further validation in well-defined patient cohorts.

This study aims to retrospectively evaluate the diagnostic utility of pleural fluid lactate and the PFL/BL ratio in a cohort of 110 patients with pleural effusion. We seek to determine their accuracy in distinguishing transudative from exudative effusions and to establish reliable cut-off values for identifying complicated parapneumonic effusions that require tube thoracostomy. Furthermore, this research will investigate the correlation between PFL and BL to clarify the autonomy of PFL as a diagnostic marker, thereby contributing to a more refined and evidence-based approach to the management of pleural effusion.

## Material and method

### Patient selection

110 patients with pleural effusion who underwent drainage between January 2016 and September 2017 were reviewed retrospectively. Ethics committee approval was obtained for the study from Harran University Faculty of Medicine, with the decision number 31 on 13.07.2017.

Patients with submassive-massive effusion causing dyspnea that did not decrease or recurring after medical treatment and had drainage indication were included in the study.

Patients with contraindications for thoracentesis were excluded from the study. The specific exclusion criteria were as follows:

- Coagulopathy: An International Normalized Ratio (INR) greater than 1.5.
- Thrombocytopenia: A platelet count below 50,000/µL.
- Anticoagulant/Antiplatelet Therapy: Recent use of anticoagulant or potent antiplatelet medications within 12—24 h prior to the procedure (e.g., heparin, warfarin, direct oral anticoagulants, clopidogrel).
- Skin Infection: Active skin or soft tissue infection at the intended puncture site.
- Patient Non-cooperation: Inability to remain still or maintain the required position due to agitation, respiratory distress, or an uncooperative state.

These criteria were established to minimize the risk of procedure-related complications, such as bleeding and infection.

### Sample collection and laboratory analysis

Samples of 50 ml of pleural fluid and 20 ml of venous blood were taken from the patients before simultaneous drainage. Pleural fluid pH (PFpH), PFL and venous BL were recorded by measuring pH and lactate levels in the same blood gas device from all samples. Pleural effusions were classified as transudates or exudates based on Light’s criteria. All effusion samples were examined for the presence of malignant cells by cytological examinations.

Patients with exudative pleural effusion were divided into 3 groups according to their diagnosis as PPE, malignant pleural effusion (MPE) and tuberculous pleurisy (TP). Patients with transudative effusion were included in the study as a single patient group.

Serum glucose, albumin, protein, LDH values were measured in Abbott Architect C 16000, and pH and lactate values of blood and pleural fluid were measured in Radiometer ABL90 Flex devices.

The effusions of patients with transudative effusion, typical PPE and MPE (no loculation was detected in thoracic computed tomography or thoracic ultrasonography) were catheterized by drainage with a 10 F pleurocan catheter. Patients with complicated PPE and MPE were drained with a 28/32F thoracic catheter. In the follow-ups, the catheters were removed when the amount of drainage decreased to 100 ml/24 h or less.

#### Statistical analysis

Data analysis was conducted with IBM SPSS Statistics software (version 22.0). Descriptive statistics for continuous variables are summarized as mean ± standard deviation, and categorical variables are reported as frequencies (n) and percentages (%). The adherence of continuous data to a normal distribution was verified with the Kolmogorov–Smirnov test. Intergroup comparisons were performed using non-parametric tests: the Mann–Whitney U test for continuous variables that deviated from normality and the Chi-square test for nominal variables. Spearman’s correlation analysis was employed to examine the relationships between continuous variables. To identify diagnostic cut-off points for parameters with significant discriminatory value, Receiver Operating Characteristic (ROC) curve analysis was utilized. For these cut-offs, performance metrics including sensitivity, specificity, positive predictive value (PPV), and negative predictive value (NPV) were derived. Statistical significance was defined a priori as a *p*-value < 0.05.

## Results

The study cohort comprised 110 patients with a mean age of 50.3 ± 19.4 years. The majority of patients were male (*n* = 67, 60.9%). Pleural effusions were more frequently located on the right side (*n* = 64, 58.2%) compared to the left (*n* = 40, 36.4%) or bilaterally (*n* = 6, 5.5%). Based on established diagnostic criteria, most effusions were classified as exudates (*n* = 90, 81.8%) rather than transudates (*n* = 20, 18.2%). Cytological examination of the pleural fluid revealed benign pleural effusions (BPE) in 80% of cases (*n* = 88) and MPE in 20% (*n* = 22) (Table 1).

**Table 1:** Baseline demographic and clinical characteristics of the study population (*n* = 110)

		n	%
Age (Mean ± SD) years	50.3 ± 19.4		
Gender			
Female		43	39.1
Male		67	60.9
Side			
Right		64	58.2
Left		40	36.4
Bilateral		6	5.5
Diagnostic Criteria			
Transudate		20	18.2
Exudate		90	81.8
Cytological Examination			
BPE		88	80
MPE		22	20

*BPE* benign pleural effusions, *MPE* malignant pleural effusion.

The PFL/BL ratio and PFL alone demonstrated the highest diagnostic utility in distinguishing exudates from transudates (*p* < 0.001 for both). whereas BL levels showed no significant discriminatory value (*p* = 0.425). Furthermore, these parameters showed significant discriminatory power among malignant, parapneumonic, and tuberculous exudative subgroups (*p* < 0.001), while BL levels remained non-discriminatory in all comparisons (Table 2).

**Table 2:** Comparison of pleural fluid biochemical parameters between transudative and exudative effusions

	Group				Subgroup		
Pleural fluid biochemistry	Transudate (Min–Max)	Exudate (Min–Max)	*p*-value	MPE	PPE	TP	*p*-value
PFpH	7.43 (7.26—7.71)	7.39 (6.3—7.49)	< 0.001	7.22 (6.36—7.43)	7.17 (6.3—7.49)	7.25 (7.13—7.44)	< 0.001
PFL	1.61 (0.3—3.7)	6.17 (1.5—22)	< 0.001	5.78 (1.7—22)	6.34 (1.5—20)	5.74 (1.6—7.9)	< 0.001
BL	1.12 (0.4—2.3)	1.33 (0.3—7.46)	= 0.425	1.26 (0.5—2.5)	1.39 (0.3—7.46)	0.86 (0.4—1.1)	0.633
PFL/BL	1.54 (0.25—4.63)	5.75 (0.47—31)	< 0.001	5.01 (1—12.22)	5.95 (0.47—31)	6.58 (3.64—9.88)	< 0.001

*PFpH* Pleural fluid pH, *PFL* Pleural fluid lactate (mmol/L), *BL* Blood lactate (mmol/L), *MPE* Malignant pleural effusion, *PPE* Parapneumonic effusion, *TP* Tuberculous pleurisy.

Data are presented as median (Min–Max). The first *p*-value column compares Transudate vs. Exudate groups, while the second *p*-value column compares the three exudative subgroups (MPE, PPE, TP).

**Fig. 1:**
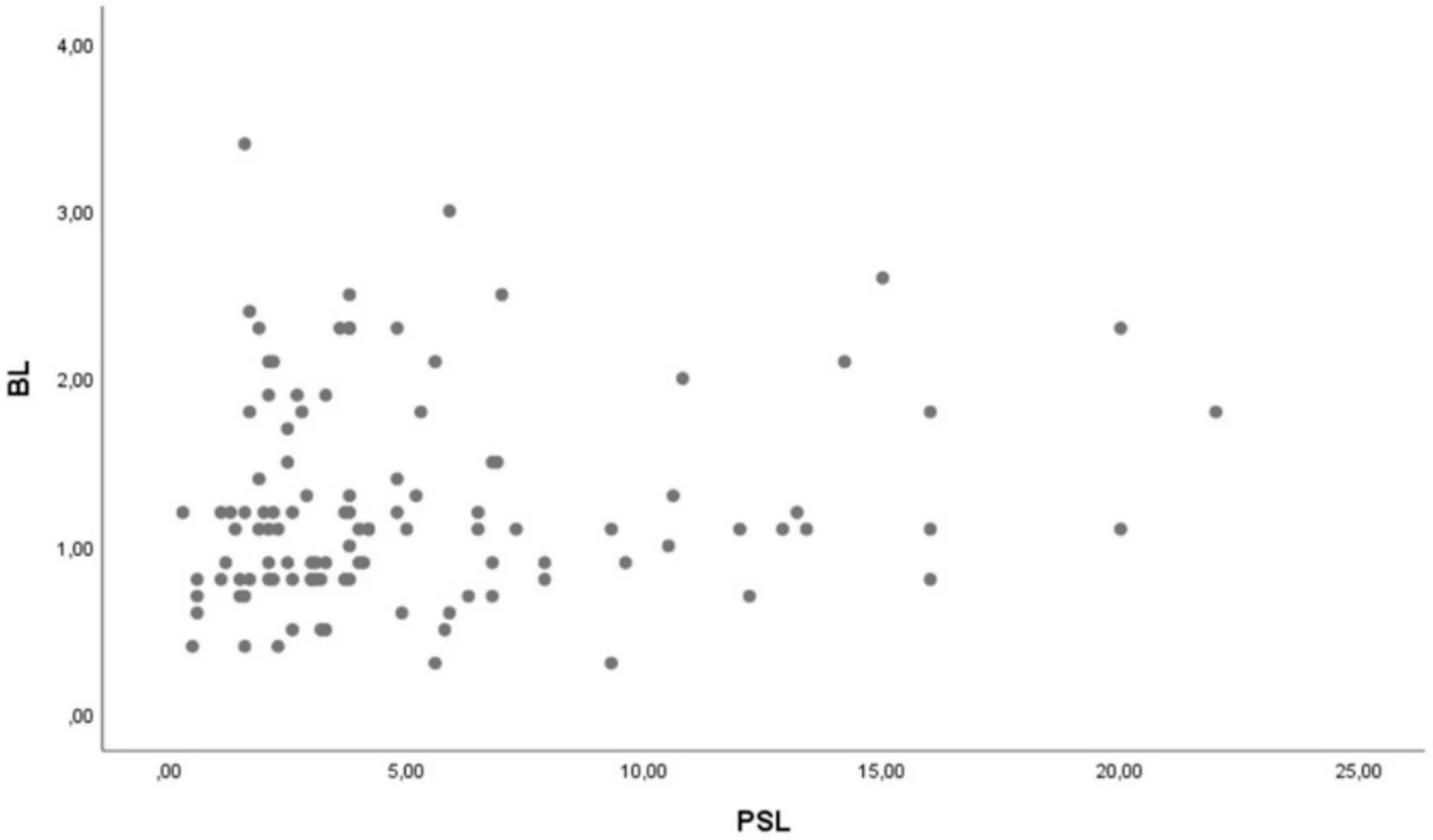
Scatter plot illustrating the correlation between PFL and BL levels. *PFL* Pleural fluid lactate (mmol/L), *BL* Blood lactate (mmol/L)

The absence of a significant correlation between PFL and BL (r = 0.163, *p* = 0.089) indicates that PFL levels were not affected by BL, a finding consistent across diagnostic categories (Fig. 1).

ROC analysis was performed to evaluate the diagnostic utility of PFL, PFL/BL ratio, and BL alone in differentiating transudative from exudative pleural effusions.

**Fig. 2:**
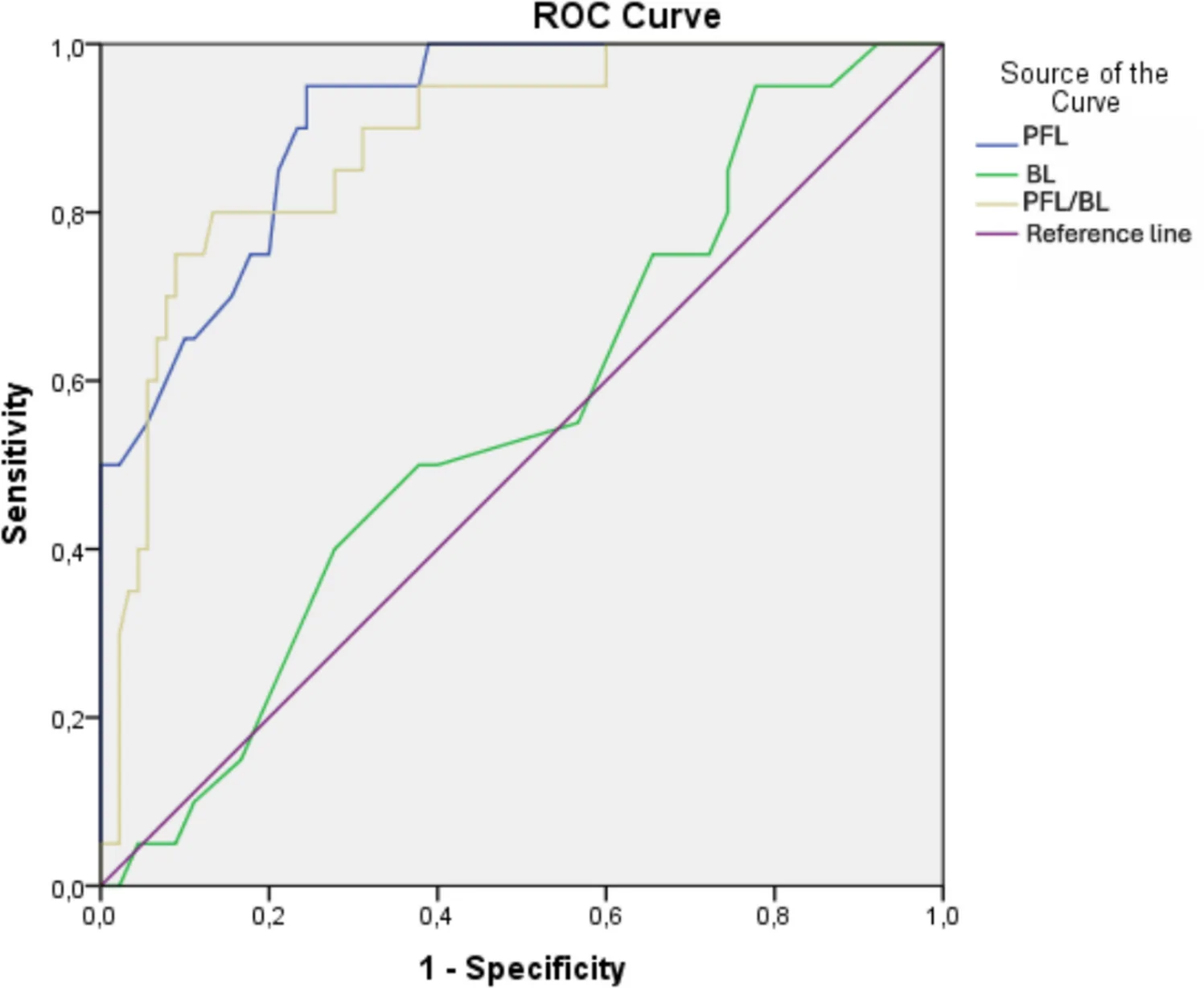
Receiver operating characteristic (ROC) curves for diagnostic parameters. *PFL* Pleural fluid lactate (mmol/L), *BL* Blood lactate (mmol/L)

The analysis revealed that PFL and the PFL/BL ratio were significant discriminators, while BL alone was not. For PFL, the optimal diagnostic cut-off was determined to be 2.85, yielding a high area under the curve (AUC) of 0.912 (95% CI: 0.854–0.971, *p* < 0.001). At this threshold, PFL demonstrated a sensitivity of 95.0% and a specificity of 75.0%. The PFL/BL ratio also showed significant diagnostic value, with a cut-off of 1.69 providing an AUC of 0.883 (95% CI: 0.806–0.961, *p* < 0.001), a sensitivity of 80.0%, and a specificity of 85.6%. In contrast, BL alone showed no significant discriminatory power (AUC = 0.557, 95% CI: 0.425–0.689, *p* = 0.427) (Table 3, Fig. 2).

**Table 3:** Diagnostic performance of PFL, PFL/BL ratio, and BL in differentiating transudative and exudative pleural effusions

Diagnostic Test	Optimal Cut-off	AUC (95% CI)	*p*-value	Sensitivity (%)	Specificity (%)	PPV (%)	NPV (%)
PFL	2.85	0.912 (0.854–0.971)	< 0.001	95.0	75.0	98.6	46.3
PFL/BL	1.56	0.883 (0.806–0.961)	< 0.001	75.0	91.1	94.3	65.0
	1.69	0.883 (0.806–0.961)	< 0.001	80.0	85.6	95.1	57.1
	1.73	0.883 (0.806–0.961)	< 0.001	80.0	85.6	95.1	55.2
BL	–	0.557 (0.425–0.689)	0.427	–	–	–	–

*AUC* Area Under the ROC Curve, *CI* Confidence Interval, *PPV* Positive Predictive Value, *NPV* Negative Predictive Value, *PFL* Pleural Fluid Lactate (mmol/L), *BL* Blood Lactate (mmol/L).

ROC analysis was performed to assess the differential diagnostic utility of PFL, PFL/BL ratio, and BL in distinguishing typical parapneumonic effusions (PPE) from complicated PPE.

**Fig. 3:**
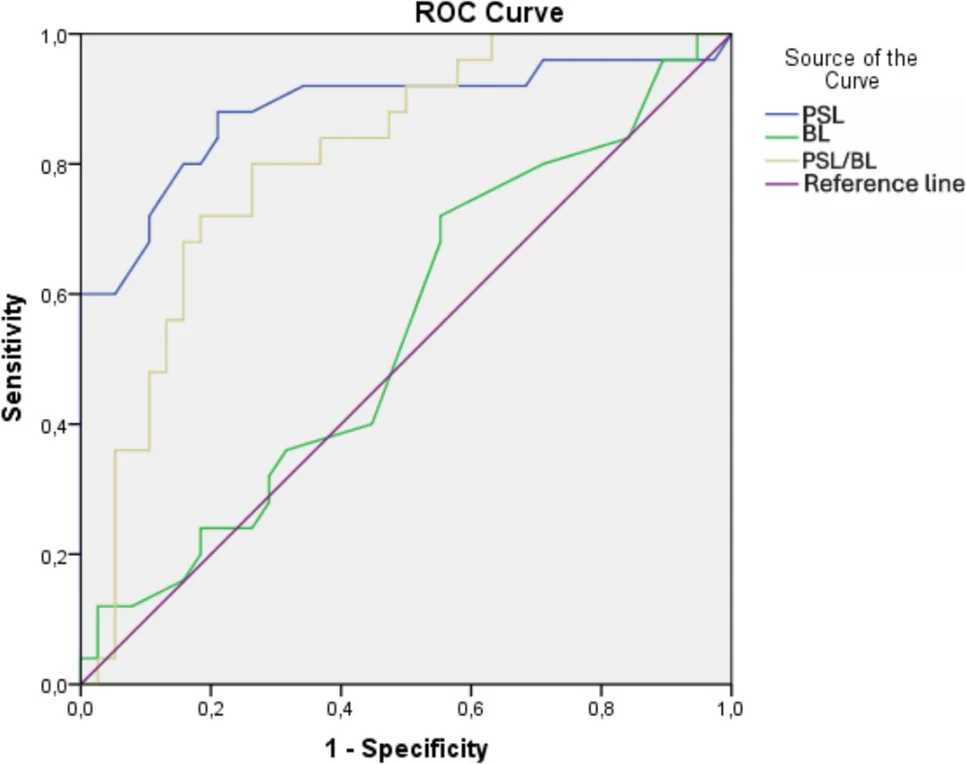
ROC curves for the differentiation of parapneumonic effusions. *PFL* Pleural fluid lactate (mmol/L), *BL* Blood lactate (mmol/L)

The analysis demonstrated that both PFL and the PFL/BL ratio were significant discriminators between these patient groups, while BL alone was not. For PFL, the optimal diagnostic cut-off was 5.45, yielding an area under the curve (AUC) of 0.883 (95% CI: 0.785–0.981, *p* < 0.001). At this threshold, PFL demonstrated a sensitivity of 80.0% and a specificity of 84.2%. The PFL/BL ratio also showed significant diagnostic value, with a cut-off of 3.77 providing an AUC of 0.809 (95% CI: 0.702–0.917, *p* < 0.001), a sensitivity of 80.0%, and a specificity of 73.7%. In contrast, BL alone showed no significant discriminatory power (AUC = 0.543, 95% CI: 0.398–0.689, *p* = 0.565) (Table 4, Fig. 3).

**Table 4:** Diagnostic performance of PFL, PFL/BL ratio, and BL in differentiating typical from complicated parapneumonic effusions (PPE)

Diagnostic Test	Optimal Cut-off	AUC (95% CI)	*p*-value	Sensitivity (%)	Specificity (%)	PPV (%)	NPV (%)
PFL	5.45	0.883 (0.785–0.981)	< 0.001	80.0	84.2	76.9	86.5
PFL/BL	3.77	0.809 (0.702–0.917)	< 0.001	80.0	73.7	66.7	84.8
BL	–	0.543 (0.398–0.689)	0.565	–	–	–	–

*AUC* Area Under the ROC Curve, *CI* Confidence Interval, *PPV* Positive Predictive Value, *NPV* Negative Predictive Value, *PFL* Pleural Fluid Lactate (mmol/L), *BL* Blood Lactate (mmol/L), *PPE* Parapneumonic Effusion.

Patients were stratified into two groups based on the pleural drainage method applied: pleurocan catheter (*n* = 66, 60%) and tube thoracostomy (*n* = 44, 40%). Analysis of pleural fluid parameters revealed that the mean levels of both PFL and the PFL/BL ratio were significantly higher in patients who underwent tube thoracostomy compared to those managed with a pleurocan catheter (*p* < 0.001 for both comparisons) (Table 5).

**Table 5:** Comparison of PFL and PFL/BL ratio between patients managed with a pleurocan catheter versus tube thoracostomy

Diagnostic Test	Drainage method	Mean	n (%)	Std. deviation	Min–max	*p*-value
PFL	Pleurocan catheter	2.83	66(60)	1.75	0.3—9.3	< 0.001
	Tube thoracostomy	9.12	44(40)	5.33	1.9—22	
PFL/BL	Pleurocan catheter	3.16	66(60)	4.06	0.25—20	< 0.001
	Tube thoracostomy	7.73	44(40)	4.81	1.36—20	

*PFL* Pleural fluid lactate (mmol/L), *BL* Blood lactate (mmol/L).

ROC analysis was conducted to evaluate the ability of PFL, PFL/BL ratio, and BL to predict the requirement for tube thoracostomy versus pleurocan catheter drainage.

**Fig. 4:**
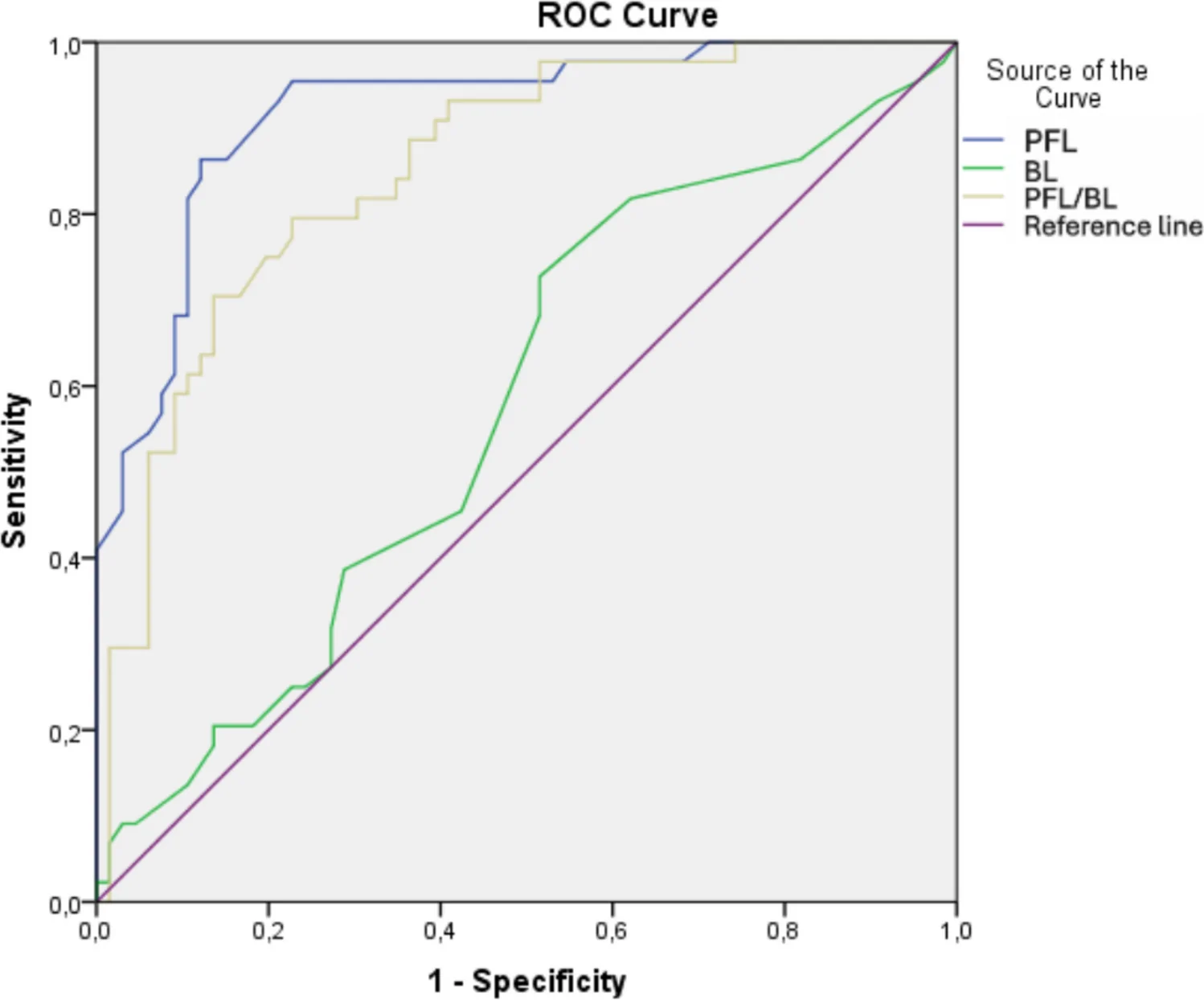
Receiver operating characteristic (ROC) curves for predicting pleural drainage method. *PFL* Pleural fluid lactate (mmol/L), *BL* Blood lactate (mmol/L)

The analysis confirmed that both PFL and the PFL/BL ratio were significant predictors of drainage type, while BL alone was not. For PFL, the optimal diagnostic cut-off was 4.05, yielding an excellent area under the curve (AUC) of 0.920 (95% CI: 0.867–0.972, *p* < 0.001). At this threshold, PFL demonstrated a sensitivity of 80.0% and a specificity of 84.2% for identifying patients requiring tube thoracostomy. The PFL/BL ratio also showed significant predictive value, with a primary cut-off of 3.77 providing an AUC of 0.853 (95% CI: 0.782–0.924, *p* < 0.001), a sensitivity of 79.5%, and a specificity of 77.3%. In contrast, BL alone showed no significant discriminatory power (AUC = 0.577, 95% CI: 0.468–0.685, *p* = 0.175) (Table 6, Fig. 4).

**Table 6:** Diagnostic performance of lactate parameters in predicting the requirement for tube thoracostomy drainage

Diagnostic Test	Optimal Cut-off	AUC (95% CI)	*p*-value	Sensitivity (%)	Specificity (%)	PPV (%)	NPV (%)
PFL	4.05	0.920 (0.867–0.972)	< 0.001	80.0	84.2	82.6	90.6
PFL/BL	3.77	0.853 (0.782–0.924)	< 0.001	79.5	77.3	70.0	85.0
	4.53	0.853 (0.782–0.924)	< 0.001	70.5	86.4	77.5	81.4
BL	–	0.577 (0.468–0.685)	0.175	–	–	–	–

*AUC* Area Under the ROC Curve, *CI* Confidence Interval, *PPV* Positive Predictive Value, *NPV* Negative Predictive Value, *PFL* Pleural Fluid Lactate (mmol/L), *BL* Blood Lactate (mmol/L).

## Discussion

This study demonstrates that PFL and PFL/BL are potent biomarkers for identifying complicated parapneumonic effusions and predicting the necessity of tube thoracostomy. Our findings indicate that these parameters provide significant diagnostic value beyond the traditional Light’s criteria, particularly in clinical scenarios where rapid intervention is critical. Specifically, elevated PFL and PFL/BL levels were found to be statistically significant predictors for surgical drainage, offering a practical and accessible tool for bedside decision-making. Consequently, incorporating lactate-based metrics into the initial evaluation of pleural infections may streamline patient management and potentially improve clinical outcomes.

Pleural effusion is a common clinical condition resulting from diverse underlying pathophysiological mechanisms, fundamentally driven by alterations in Starling forces that disrupt the balance between pleural fluid formation and absorption [1]. Accurate and timely differentiation between transudative and exudative effusions remains the cornerstone of diagnostic evaluation, as it directly guides subsequent etiological investigation and therapeutic decision-making.

In previous studies, the reported proportion of transudative pleural effusions has ranged from 25 to 30%, while exudative effusions constitute approximately 70–75% of cases. Teixeira et al. reported transudative and exudative effusion rates of 29.8% and 70.2% [10], respectively, whereas Coşar et al. [11] found similar distributions in their cohort. In contrast, our study demonstrated a lower proportion of transudative effusions (18.2%). This discrepancy is likely attributable to our study design, as only patients requiring pleural drainage were included, inherently selecting for more clinically significant and predominantly exudative effusions.

One of the most notable findings of the present study is the absence of a significant correlation between PFL and BL levels. Although previous investigations suggested that elevated systemic lactate might influence pleural lactate concentrations—particularly in critically ill patients—our data demonstrate that PFL behaves independently of BL [6]. The lack of correlation (r = 0.163, *p* = 0.089) across the entire cohort and within diagnostic subgroups supports the hypothesis that PFL primarily reflects localized metabolic and inflammatory activity within the pleural space rather than systemic lactate dynamics. This finding reinforces the diagnostic autonomy of PFL and aligns with studies reporting minimal influence of systemic lactate on pleural fluid biochemistry.

Our results demonstrate that both PFL and the PFL/BL ratio exhibit high diagnostic accuracy in distinguishing transudative from exudative pleural effusions. A PFL cut-off value of 2.85 mmol/L yielded a sensitivity of 95.0% and a specificity of 75.0%, outperforming several previously proposed thresholds. Porta et al. [6] reported a lower cut-off value of 1.9 mmol/L, with corresponding sensitivity and specificity of 80% and 78%, respectively. Similarly, Santotoribio et al. identified a PFL threshold of 2.1 mmol/L with moderate diagnostic performance [7]. Compared with these studies, our higher cut-off value demonstrated superior overall diagnostic performance, as reflected by a higher area under the ROC curve (AUC = 0.912). These differences may be explained by variations in patient selection, disease severity, and analytical techniques.

The PFL/BL ratio also emerged as a robust diagnostic parameter. In our cohort, a cut-off value of 1.73 achieved a sensitivity of 80.0% and a specificity of 85.6% for identifying exudative effusions, exceeding the diagnostic performance reported in prior studies [6, 7]. Importantly, BL alone showed no discriminatory value, further underscoring that pleural lactate-related parameters are superior indicators of pleural pathology.

According to Santotoribio et al. in the same study, the importance of PFL levels in 30 patients with PPE in the diagnosis and treatment of PPEs was evaluated [7]. Ten of the patients were typical and 20 were complicated PPE. When the cut-off value of PFL was 10.2 mmol/L, complicated PPE was distinguished from typical PPE; They stated that the sensitivity was 45% and the specificity was 90%. However, contrary to the study, the cut-off value of PFL was 5.45, with a smaller sensitivity of 80% and a specificity of 84.2%. This was attributed to the higher number of patients in our study and the fact that PFL was studied on different devices.

The observed elevation of PFL in exudative effusions without a proportional decrease in pleural fluid pH warrants further consideration. While acidic pleural fluid is characteristic of advanced inflammatory states, not all exudative effusions are associated with acidosis [12–15]. In a study on lactic acidosis, they evaluated the causes of increased blood lactic acid [16]. In cases where cellular metabolism increases, glycolysis increases to initiate the Krebs cycle in the cell. Type B hyperlactatemia occurs without acidosis [17–20]. Small amounts of pyruvic acid do not enter the Krebs cycle. Pyruvate is converted to lactate by lactate dehydrogenase to maintain the NAD +/NADH ratio during accelerated glycolysis. For this reason, in cases where metabolism increases, pyruvate elevation occurs along with lactate elevation [21–23]. On the other hand, the high lactate level in exudative effusions is thought to be related to the activation of immune system cells in inflammatory fluids [21, 24]. Studies have reported that lactate causes signaling processes for macrophage change [25, 26]. Additionally, bacterial metabolism—especially under anaerobic conditions—contributes to elevated lactate levels, further supporting the role of PFL as a marker of localized inflammatory and infectious activity [27, 28].

While BPEs constituted 88% of pleural effusions in one study [29], this rate was 52% in patients who underwent thoracoscopy for pleural effusion in another study [30]. In our article, it was 20% of patients with MPE and 80% of patients with BPE.

From a therapeutic perspective, our findings demonstrate a strong association between elevated PFL levels and the need for tube thoracostomy. Patients managed with tube thoracostomy exhibited significantly higher PFL and PFL/BL ratio values compared with those treated using a pleurocan catheter. ROC analysis identified a PFL cut-off value of 4.05 mmol/L as a reliable predictor for tube thoracostomy requirement, with a sensitivity of 80.0% and specificity of 84.2%. This threshold is lower than previously suggested values but is consistent with clinical practice patterns guided by Light’s classification, particularly in borderline complicated PPE where early tube thoracostomy may prevent disease progression [7].

Our findings are further supported by recent literature, including pediatric and adult cohorts, demonstrating the utility of PFL in differentiating infectious pleural effusions and guiding therapeutic decisions [8, 9, 31]. Notably, a 2023 study by Johansson et al. corroborated the high sensitivity and specificity of PFL in distinguishing simple from complicated parapneumonic effusions, reinforcing the contemporary relevance of our results [32].

Taken together, the alignment between our findings and those of Johansson et al. provides strong external validation for the use of pleural fluid lactate in pleural effusion management. Our study expands upon their work by demonstrating that PFL not only identifies complicated parapneumonic effusions but also reliably predicts the need for tube thoracostomy, thereby bridging diagnostic assessment and therapeutic decision-making.

Our findings align closely with the contemporary surgical framework for pleural infection outlined by Mastromarino et al., which emphasizes early identification of disease severity and timely escalation of invasive management to prevent treatment failure. While current algorithms rely primarily on pleural pH, glucose, imaging features, and clinical response, our results demonstrate that PFL provides an additional, objective marker of localized inflammatory and metabolic activity. Notably, PFL was independent of systemic BL and showed high diagnostic accuracy in distinguishing typical from complicated parapneumonic effusions, as well as in predicting the need for tube thoracostomy. These findings support the concept that elevated PFL reflects advanced pleural infection biology, consistent with the fibrinopurulent stage described by Mastromarino et al., where conservative drainage often fails. Incorporation of PFL into pleural fluid assessment may therefore enhance early risk stratification and refine surgical decision-making, facilitating timely escalation to more effective drainage strategies and potentially reducing delays to definitive intervention [33].

## Conclusion

In this study, PFL and the PFL/BL ratio demonstrated high accuracy in differentiating transudative from exudative effusions, while BL alone showed no diagnostic utility. The absence of a significant correlation between pleural and systemic lactate levels supports the concept that pleural fluid lactate reflects localized inflammatory and metabolic activity rather than systemic physiology.

Importantly, elevated PFL levels were associated with disease severity and the need for more invasive pleural drainage. PFL effectively distinguished typical from complicated parapneumonic effusions and reliably predicted the requirement for tube thoracostomy, highlighting its potential role as a decision-support tool in surgical practice.

Incorporation of PFL into routine pleural fluid analysis may assist surgeons and clinicians in early risk stratification and optimal drainage selection, particularly in borderline or evolving parapneumonic effusions. Prospective multicenter studies are needed to validate standardized cut-off values and to define the role of pleural fluid lactate within contemporary pleural disease management algorithms.

## Limitations

Several limitations of this study should be acknowledged. Its retrospective, single-centre design may have introduced selection bias and limits external validity. Only patients requiring pleural drainage were included, which likely resulted in an overrepresentation of exudative and clinically advanced effusions, potentially influencing diagnostic performance estimates of PFL. In addition, PFL measurements were obtained using a blood gas analyser, and inter-device variability may affect the generalisability of the proposed cut-off values. Microbiological yield was limited, precluding pathogen-specific analyses and correlation with disease severity. Although PFL was strongly associated with drainage strategy, surgical endpoints such as progression to VATS or open decortication, postoperative outcomes, and long-term recurrence were not systematically evaluated. Finally, the absence of prospective validation limits causal interpretation, and multicentre prospective studies are warranted to confirm these findings and define the role of PFL within contemporary pleural disease management algorithms.

## Data Availability

Data is available upon request to the corresponding author.
